# Altered Decision-Making under Risk in Obesity

**DOI:** 10.1371/journal.pone.0155600

**Published:** 2016-06-03

**Authors:** Juan F. Navas, Raquel Vilar-López, José C. Perales, Trevor Steward, Fernando Fernández-Aranda, Antonio Verdejo-García

**Affiliations:** 1 Department of Experimental Psychology, University of Granada, Granada, Spain; 2 Red de Trastornos Adictivos, and Brain, Mind and Behaviour Research Center (CIMCYC),University of Granada, Granada, Spain; 3 Department of Personality, Assessment and Psychological Treatment,University of Granada, Granada, Spain; 4 Department of Psychiatry, Bellvitge University Hospital-IDIBELL, Barcelona, Spain; 5 CIBER Fisiopatología de la Obesidad y Nutrición (CIBERObn), Instituto Salud Carlos III (ISCIII), Barcelona, Spain; 6 School of Psychological Sciences & Monash Institute of Cognitive and Clinical Neurosciences, Monash University, Melbourne, Australia; National Scientific and Technical Research Council (CONICET)., ARGENTINA

## Abstract

**Background:**

The negative consequences of energy dense foods are well known, yet people increasingly make unhealthy food choices leading to obesity (i.e., risky decisions). The aims of this study were: [1] to compare performance in decision-making tasks under risk and under ambiguity between individuals with obesity, overweight and normal weight; [2] to examine the associations between body mass index (BMI) and decision-making, and the degree to which these associations are modulated by reward sensitivity.

**Methods:**

Seventy-nine adults were recruited and classified in three groups according to their BMI: obesity, overweight and normal-weight. Groups were similar in terms of age, education and socio-economic status, and were screened for comorbid medical and mental health conditions. Decision-making under risk was measured via the Wheel of Fortune Task (WoFT) and decision-making under ambiguity via the Iowa Gambling Task (IGT). Reward sensitivity was indicated by the Sensitivity to Punishment and Sensitivity to Reward Questionnaire (SPSRQ).

**Results:**

Individuals with obesity made riskier choices in the WoFT, specifically in choices with an expected value close to zero and in the propensity to risk index. No differences were found in IGT performance or SPSRQ scores. BMI was associated with risk-taking (WoFT performance), independently of reward sensitivity.

**Conclusions:**

Obesity is linked to a propensity to make risky decisions in experimental conditions analogous to everyday food choices.

## Introduction

In Western societies we are constantly challenged by food choices that are attractive but not necessarily healthy. Many of these food choices can be regarded as risky, as explicit information about their negative consequences is available (e.g., saturated fats). Other food choices are ambiguous, as their potential negative consequences are unknown, or difficult to gauge based on available information (e.g., processed meat). The decision-making processes underpinning these choices are critical to the understanding of obesity, since excess weight in current societies is mostly driven by choices involving frequent selection of highly palatable food despite negative health consequences [1]. This pattern of choice can be captured by performance on laboratory tasks of decision-making (see [2] for a review).

In cognitive neuroscience, a distinction is made between decision-making tasks under ambiguity and under risk. In ambiguity conditions, the magnitude and the probability of reward are initially unknown to the learner and only revealed through feedback on one’s choices. In risk conditions, this information is made explicit [3]. Most research on obesity has been conducted using tasks of decision-making under ambiguity (e.g. the Iowa Gambling Task, IGT), and has shown that individuals with obesity, compared to normal weight controls, have a preference for superficially high, short-term rewards, despite subsequent long-term losses [4–7]. However, the conclusions of these studies are still debated, as the obesity samples examined have not always been matched to comparison groups in potentially relevant confounders. Specifically, few studies have systematically controlled for sex, education, and/or comorbid conditions. When these variables are factored in, differences between groups tend to decrease or even vanish [6, 8].

Critically, in current societies, the potential negative consequences of eating certain foods are accessible via nutrition facts [9], and hence tasks of decision-making under risk are more ecologically valid. In spite of its relevance, decision-making under risk is under-examined among excess weight populations. Anderson & Mellor (2008) [10]showed that individuals with overweight and obesity make riskier decisions compared to healthy-weight controls in a lottery choice task; however, the implications of their findings were limited by the fact that BMI was assessed via self-report. More recently, Chamberlain et al. (2015) [11] showed that individuals with obesity display less modulation of behavior as a function of risk in the Cambridge Gamble Task; however, part of their obesity sample had relevant comorbidities, such as gambling, which may have again confounded the result.

A key (although often neglected) construct linked to decision-making under ambiguity and risk is reward sensitivity. Gray's Reinforcement Sensitivity Theory posits that the behavioral approach system directs behavior towards appetitive stimuli that provide immediate compensation (e.g. appetizing food) and is represented by the trait of “sensitivity to reward” (SR) [12]. Theoretically, SR can have a significant effect both in risk- and ambiguity-based tasks. In the first type of tasks (risk), people with high SR has shown to be more prone to tolerate a smaller probability of reward (and thus a higher risk), if such reward is subjectively overvalued [13]. In the second type of tasks (ambiguity), high SR can impact the subjective value of positive versus negative feedback, and hence bias learning towards superficially attractive choices [14].

In the field of obesity and eating behavior, neuroimaging studies have linked SR to the neural orchestration of food choices [15,16] and personality research has shown an inverted U-shape relationship between BMI and SR, with obesity individuals showing lower SR than both normal-weight and overweight individuals [17]. However, this link has not been established for objective laboratory measures of decision-making. This is relevant, as people with higher SR, as noted above, perform more poorly on some decision-making tasks [18], and thus SR could contribute to decision-making anomalies underlying food-choice and leading to obesity [19].

In the present study, we compare the performance of three groups defined according to BMI (normal weight, overweight, and obesity) in tasks of decision-making under risk and decision-making under ambiguity. We had two main aims: (i) to establish the relationship between BMI and decision-making under risk (in the WoFT) and ambiguity (in the IGT), after controlling for relevant confounders (socio-demographic factors, comorbidities, and sex); (ii) to determine the extent to which SR associates with decision-making under risk and ambiguity in the context of overweight and obesity.

Based on the conceptual similarities between food-choice tasks and risk-based tasks, we expect obese participants to show riskier decisions than overweight and healthy weight participants in the WoFT (Hypothesis 1). In view of the abovementioned evidence limiting the interpretation of previous studies that have shown differences between obese and control groups in ambiguity-based tasks, we expect no effect attributable to BMI on the IGT (Hypothesis 2). In addition, we expect SR exerts an effect on both the IGT and the WoFT measures (Hypothesis 3). Given the dearth of research linking SR to decision-making performance in the context of excess weight, we did not specify the directionality of this latter hypothesis.

## Methods

### Participants

Seventy-nine adults classified in three groups according to their BMI: normal-weight (BMI>18<25; n = 38), overweight (BMI>25<30; n = 21) and obese (BMI>30; n = 20) [20]. In order to achieve sample representativeness, participants were recruited through a variety of sources, including local newspapers, social media, and hospitals and clinics. The inclusion criteria were (1) being between 18 and 45 years of age (to minimize the impact of ageing in decision-making performance); and (2) having a BMI between 18 and 40, as indicated by an automated (Tanita) scale. The exclusion criteria were as follows: (1) having comorbid medical conditions associated with obesity, such as diabetes, fatty liver disease and hypertension indicated by blood count and blood pressure tests supervised by accredited nursing staff; (2) having current neurological or mental health disorders, indicated by clinical interviews conducted by accredited psychologists. Sociodemographic characteristics are displayed in Table 1.

**Table 1:** Demographical and clinical descriptive data.

	NW	OW	OBS	Test Statistics	*p*
	n/(%)	n/(%)	n/(%)		
Sex					
Female	22/(57.89)	11/(52.38)	11/(55)		
Male	16/(42.11)	10/(47.62)	9/(45)		
Total	38	21	20	*χ*^*2*^ (2,76) = 0.91	NS
	Mean (SD)	Mean (SD)	Mean (SD)		
Age	33.18 (6.59)	35.00 (6.31)	32.15 (5.96)	F(2,76) = 1.072	NS
Education (years)	18.29 (3.78)	17.86 (3.58)	16.95 (4.01)	F(2,76) = 0.821	NS
Monthly Income	%	%	%		
<600 €	21.62	9.52	10.53		
601–1000 €	10.81	9.52	15.79		
1001–1500 €	21.62	28.57	21.05	*χ*^*2*^ (2,75) = 10.878	NS
1501–2000 €	18.92	14.29	15.79		
2001–2500 €	8.11	9.52	31.58		
>2500 €	18.92	28.57	5.26		
BMI	22.21 (1.70)	27.34 (1.59)	33.50 (2.60)	F(2,76) = 224.85	p = <0.001
Fat%	19.98 (5.82)	28.23 (7.56)	35.16 (8.73)	F(2,72) = 28.799	p = <0.001
SR	10.10 (3.98)	10.14 (3.81)	10.40 (6.02)	F(2,76) = 0.029	NS
SP	9.89 (4.40)	12.14 (5.34)	10.35 (5.38)	F(2,76) = 1.451	NS

Note: Degrees of freedom differ due to missing data. Abbreviations: NW = Normal weight; OW = Overweight; OBS = Obesity; SR = Sensitivity to reward; SP = Sensitivity to punishment; NS = Non significant.

### Measures

#### Anthropometric measures

BMI was calculated for each participant using the ratio of weight in kilograms divided by the square of height in meters. Weight and body fat percentage were recorded through the use of a digital scale and body composition analyzer (Tanita DC-430U).

#### Trait measures

*Sensitivity to Punishment and Sensitivity to Reward Questionnaire*. (SPSRQ, [21]). This yes/no questionnaire assesses reward and punishment sensitivity (SR/SP). It comprises 24 items for each scale. The Spanish version of this scale has demonstrated good internal consistency (SP, α = .81 to .83; SR, α = .73 to .76), and acceptable reliability and validity. Due to the main aim of this study, we focused on SR scale. Nonetheless, SP was also taken into account in statistical analyses.

#### Cognitive tasks of decision-making

*The Wheel of Fortune Task* (WoFT) [22]: The WoFT is a computerized choice task in which the probability and magnitude of gains and losses are explicitly displayed in each trial. Participants were instructed to choose between two wheel-of-fortune roulettes, each divided into eight segments. Each segment of the roulette displayed the amount of points participants could win or lose. For example, a roulette containing six segments with the text "+80" and two segments with the text "-20" have a .75 probability of winning 80 points and a .25 probability of losing 20 points; its expected value (EV = probability sum of the product for the X variable winning and losing outcome) would be 55.

The task was composed of 10 types of trials: Eight of them require a choice between a control roulette and a high-risk roulette wheel (Table 2). The control wheel consisted of 4 "+10" segments and 4 "-10" segments (P = .5 to win 10 points; P = .5 to lose 10 points, EV = 0). The high-risk wheel offered a low (P = .25) or high (P = .75) probability of winning; medium (20 points) or high reward (80 points); and a high (-80) or medium (-20) loss. Henceforth in the text, these trials are labeled with the high probability outcome, followed by the low probability outcome in parentheses; for example, "+80 (-20)" denotes high probability of a high reward and low probability of a medium loss. The EV varies from positive to negative values across the trial types (Table 2). ΔEV is the difference between the EV of the high-risk and the control wheels. The other two trial types consisted of conditions of gains only (control wheel: P = 1 to earn an average of 40 points vs. high-risk wheel: P = .5 to win a large reward of 80 points) or losses only (control wheel: P = 1; -40 points; high-risk wheel: P = .5; -80). The ΔEV of both is 0, and hence choosing the high-risk wheel is regarded as a propensity-to-risk index.

**Table 2:** Risky trials in the Wheel of Fortune Task.

	Trial Type	P Win	Magnitude	P Loss	Magnitude	ΔEV
1	**-80(+20)**	.25	20	.75	-80	-55
2	**-80(+80)**	.25	80	.75	-80	-40
3	**-20(+20)**	-25	20	.75	-20	-10
4	**+20(-80)**	.75	20	.25	-80	-5
5	**-20(+80)**	.25	80	.75	-20	5
6	**+20(-20)**	.75	20	.25	-20	10
7	**+80(-80)**	.75	80	.25	-80	40
8	**+80(-20)**	.75	80	.25	-20	55
**Only gains**		.5	80	.5	0	0
**Only losses**		.5	0	.5	-80	0

The first value in the trial type refers the high probability outcome and the value in parentheses refers the low probability outcome. Trials 1 to 8 in the control roulette offer small wins and losses (+10 and -10 points) with a probability (P) of .5. The gains- and losses-only wheels offer a determined average of wins or losses (+40 or -40; P = 1) respectively. ΔEV = difference between the expected values of the risky and control gambles.

The task consisted of a total of 56 trials conducted in 3 blocks. Each test consisted of 3 phases: (1) both roulette wheels appeared on the screen and the participant were instructed to choose one of them; (2) the selected wheel was placed in the middle of the screen and different segments of the roulette wheel spun for a period of between 5 and 5.5 seconds; (3) the wheel stopped and the final result was displayed for 2 seconds. At the end of each block, information was given to the participant on the number of points accumulated in the block. The dependent variables for our analysis were the proportion of risky roulette wheel choices in each condition.

*The Iowa Gambling Task* (IGT, [23]). This is a computer task that required participants to choose 100 cards from four card decks (A, B, C, D). Participants were instructed to select cards to earn as much money as possible and initially, explore the outcome of the different card decks under ambiguity to eventually choose stable reward where the risks and benefits are more explicit. Unbeknownst to them, decks A and B are considered 'good decks' because they contain the most cards with modest wins and small losses, leading to net gains. On the other hand, C and D decks are 'bad decks' that combine large wins with even larger losses that led to net losses. The dependent variable for our analysis was the mathematical difference between the number of cards chosen from the advantageous decks and the number of cards chosen from the disadvantageous decks

### Procedure

Following recruitment adverts, 503 applications were initially received via email. Requests were followed-up in order of receipt via telephone interviews to screen for eligibility until completion of the required sample size according to power analyses (n = 80). Seventeen participants were excluded due to mental health disorders; 14 due to medical conditions; 38 due to self-reported BMI above 40 or below 19; and 12 for not meeting the age criteria. Eighty-two participants were successfully screened and invited to participate. The inclusion criteria were confirmed in a face-to-face session via biomedical tests (blood count, blood pressure) and a clinical interview. Three additional participants were excluded from the study at this stage (final sample N = 79).

The first experimental session included objective assessments of weight, height and body fat measures, along with sociodemographic information. In a second session, the WoFT, IGT and SPSRQ were administered via desktop computers located at the Neuroeconomics lab of the Faculty of Economics. Due to technical problems in these computers the IGT data for one participant was lost. Participants received between 5 to 10 Euros as reimbursement for cognitive assessments, depending on performance on the tasks.

### Statistical analysis

Statistical analyses were conducted in SPSS 22. The SPSS dataset is included as S1 File. The significance threshold was set at p<0.05. We used general linear model’s analyses of variance (ANOVAs) to compare the groups in age, education, adiposity, and Sensitivity to Reward and Punishment (SR/SP) measures. Chi-square tests were used to compare sex and socio-economic status breakups. To examine group differences in performance on tasks of decision-making, a MANCOVA was used for the WoFT and an MANCOVA for the IGT, using SR and SP as covariates. Based on previous research that pointed out an influence of sex on IGT [8]we replicated all analyses controlling for this variable. These analyses were carried out in two ways: 1) incorporating sex as a covariate to the current analyses, and 2) considering sex as a second between subject-factor, to be considered along with BMI group (NW, OW, and OBS), and the sensitivity to reward and sensitivity to punishment as covariates. In none of these analyses sex explained away or altered the main effects of BMI groups reported below. Due to the main effects of BMI group on decision-making performance remained unchanged after controlling for sex, we henceforth only report uncorrected results.

Complementarily, stepwise linear regressions were conducted to determine the effect of SR/SP and BMI on WoFT and IGT performance, controlling for socio-demographic variables.

### Ethical standards

All procedures contributing to this work comply with the ethical standards of the Helsinki Declaration of 1975, as revised in 2008. In addition the Ethics Committee for Human Research of the University of Granada approved the study and all participants signed informed consent.

## Results

### Socio-demographic, clinical characteristics and SR/SP

Results are displayed in Table 1. As expected, normal-weight, overweight and obese groups differed in BMI and body fat percentage. We did not find significant group differences between in sex, age, education, monthly income, or SR/SP.

### Wheel of Fortune Task (WoFT)

**Fig 1:**
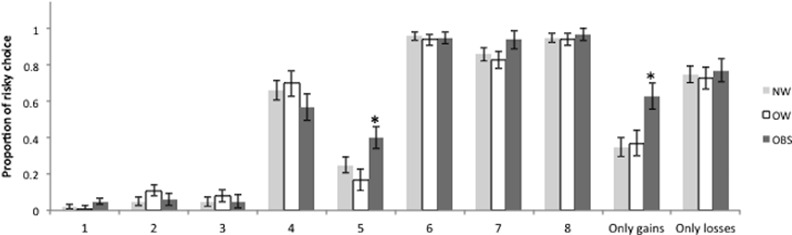
Proportion of risky choices per condition on the Wheel of Fortune Task. 1 = -80(+20); 2 = -80(+80); 3 = -20(+20); 4 = +20(-80); 5 = -20(+80); 6 = +20(-20); 7 = +80(-80); 8 = +80(-20). Abbreviations: NW = Normal weight; OW = Overweight; OBS = Obesity. *Significant difference

The proportions of risky choices for each group are shown in Table 3 and Fig 1. Participants with obesity made more risky choices in -20 (+80) trials, and in the gains-only condition compared to overweight and normal weight. MANCOVA results showed that SR and BMI had a significant effect on the proportion of risky choices (Wilks 'λ = 0.718, *p*<0.05, η^2^ = 0.282, and Wilks' λ = 0.618, *p*<0.05, η^2^ = 0.214, respectively). SR had a significant effect on trials -80 (+20); -80 (+80); -20 (+20); -20 (+80) and gains-only (Table 4). The effect of BMI was only significant on trials -20 (+80) and gains-only (see Table 4). SP did not have a significant effect on risky choices.

**Table 3:** Proportion of risky choices per condition in the Wheel of Fortune Task.

	-80	-80	-20	+20	-20	+20	+80	+80	Only	Only
	(+20)	(-80)	(+20)	(-80)	(+80)	(-20)	(-80)	(-20)	gains	losses
**NW**	0.020	0.053	0.046	0.657	0.253	0.961	0.862	0.954	0.352	0.750
**(N = 38)**	(0.068)	(0.119)	(0.182)	(0.330)	(0.279)	(0.124)	(0.207)	(0.163)	(0.322)	(0.293)
**OW**	0.011	0.107	0.083	0.702	0.167	0.941	0.833	0.941	0.363	0.726
**(N = 21)**	(0.055)	(0.169)	(0.183)	(0.350)	(0.195)	(0.135)	(0.278)	(0.175)	(0.335)	(0.297)
**OBS**	0.050	0.063	0.050	0.563	0.400*	0.950	0.938	0.975	0.625*	0.770
**(N = 20)**	(0.103)	(0.160)	(0.103)	(0.305)	(0.328)	(0.174)	(0.179)	(0.077)	(0.314)	(0.252)

Mean score (Standard deviation). Abbreviations: NW = Normal weight; OW = Overweight; OBS = Obesity.

* Significant difference between groups.

**Table 4:** Influence of sensitivity to reward, sensitivity to punishment and body mass index on the Wheel of Fortune Task.

Trial Type	SR			SP			BMI		
	*F* (1,74)	*p*	η^2^	*F* (1,74)	*p*	η^2^	*F* (2,74)	*p*	η^2^
**-80 (+20)**	10.130	0.002	0.120	1.944	NS	--	1.755	NS	--
**-80 (+80)**	15.192	< .001	0.170	1.860	NS	--	0.800	NS	--
**-20 (+20)**	6.793	0.011	0.084	2.699	NS	--	0.726	NS	--
**+20 (-80)**	0.602	NS	--	0.073	NS	--	0.942	NS	--
**-20 (+80)**	11.535	0.001	0.134	0.476	NS	--	4.248	0.018	0.103
**+20 (-20)**	1.075	NS	--	0.337	NS	--	0.093	NS	--
**+80 (-80)**	0.036	NS	--	0.055	NS	--	1.152	NS	--
**+80 (-20)**	0.298	NS	--	0.756	NS	--	0.204	NS	--
**Only gains**	4.601	0.035	0.058	0.717	NS	--	5.161	0.008	0.122
**Only losses**	0.579	NS	--	0.010	NS	--	0.102	NS	--

Abbreviations: SR = sensitivity to reward; SP = sensitivity to punishment; BMI = body mass index

Regression models showed that SR was the only predictor of performance in trials -20 (+80), after controlling for age, sex, education, BMI and SP (β = 0.353, *p* = 0.001, F = 10.96; η^2^ = 0.113). Conversely, BMI was a better predictor than SR for the gains-only condition, after controlling for age, sex, education and SP (β[BMI] 0.328, β[SR] = 0.238, *p* = 0.001, F = 7.54; η^2^ = 0.144).

### Iowa Gambling Task (IGT)

There were no differences between groups in the number of disadvantageous choices in the IGT (Table 5). MANCOVA analysis showed that SR, SP and BMI did not have a significant effect on IGT performance (SR: F (1,73) = 0.69, *p*> 0.05, η^2^ = 0.001; SP: F (1, 73) = 0.154, *p*> 0.05, η^2^ = 0.002; BMI: F (2,73) = 0.757, *p*> 0.05, η^2^ = 0.020).

**Table 5:** Between groups differences in the number of disadvantageous choices in The Iowa Gambling Task (IGT).

	NW	OW	OBS	Test Statistics	
	Mean (SD)	Mean (SD)	Mean (SD)	F(2,75)	
Block 1	-3.32 (7.18)	-4.20 (5.11)	-4.90 (6.41)	0.408	NS
Block 2	1.53 (6.42)	1.70 (4.37)	1.90 (7.50)	0.024	NS
Block 3	3.58 (7.43)	4.30 (7.98)	6.50 (6.96)	1.016	NS
Block 4	3.32 (8.70)	4.00 (8.92)	8.10 (8.17)	2.108	NS
Block 5	4.79 (9.59)	5.60 (9.30)	7.40 (8.68)	0.517	NS
Total	9.89 (27.34)	11.40 (26.35)	19.00 (27.49)	0.765	NS

Abbreviations: NW = Normal weight; OW = Overweight; OBS = Obesity; NS = Non significant.

## Discussion

The overarching aim of this study was to establish the association between obesity and decision-making under risk and ambiguity. We showed that individuals with obesity display riskier decision-making under risk compared to individuals who are overweight or have normal weight. Specifically, propensity-to-risk choices were predicted by BMI, independently of trait sensitivity to reward, and after controlling for relevant confounders. Conversely, there were no differences between excess-weight (overweight and obesity) and normal-weight individuals on decision-making under ambiguity.

In the WoFT (risk task), individuals with obesity made more risky choices specifically in conditions that did not lead to disproportionately large losses, confirming our first hypothesis. Critically, it has been proposed that this type of risky choices is akin to real-life food choices, in which the potential risk (e.g., caloric content) is estimated on a meal-by-meal basis, and thus the negative consequences are never exceedingly large [24]. Performance in the remaining trials indicates that obese individuals adjust their decision-making to expected values in a similar way to overweight and normal-weight individuals. This decision-making profile suggests that obese individuals can adequately evaluate the consequences of choices, but are prone to make risky choices when the anticipated reward is equal or slightly higher than the expected loss. This interpretation is supported by functional neuroimaging studies that have shown that individuals with obesity display increased responsivity of the midbrain (part of the brain’s reward system) and decreased responsivity of the insula (brain’s risk-processing system) during anticipation when making this type of risky choices [25,26].

Relatedly, SR had a significant and independent effect on decision-making under risk. This results is in fitting with previous reports [13] suggesting that risky decisions can result both from decreased uncertainty aversion and/or from overvaluation of ensuing reward [27]. Nonetheless, BMI groups did not differ in SR, and the effect of group (or BMI, considered as a continuous factor in regression analyses) was not explained away or diminished when controlling SR. In other words, BMI *per se* had an impact on the WoFT, independently or SR. The potential neuropsychological mechanisms underlying the effect of SR are undoubtedly interesting, but remain beyond the aims of the present study.

We found no differences between BMI groups on decision-making under ambiguity as indicated by the IGT, confirming our second hypothesis. Previous studies have shown that adults with obesity, compared to individuals with normal weight, exhibit poorer performance in this task [4–7]. However, Davis *et al*. (2010) [6] and Koritzky et al. (2012) [8] found that this difference disappeared when relevant confounders were factored in (years of education, sex and clinical comorbidities). Indeed, a recent systematic review of cognitive studies in obesity have emphasized the poor consistency of the research findings within this domain [28]. Lack of consistency has been ascribed to samples including comorbid conditions, and systematic differences in socio-demographic variables, such as education and socioeconomic status [6,28]. Since the present study adequately controlled for these variables, we interpret that there is no meaningful association between obesity and decision-making under ambiguity.

The study has important strengths. First, it is the first study to test decision-making under risk and under ambiguity in obesity, while also controlling for the effect of sensitivity to reward and relevant confounders. Second, it differentiates between obesity and overweight, which are often clumped together in the obesity literature. In addition, the study may have relevant clinical implications. Currently available weight loss interventions emphasize educational aspects (i.e., explain the consequences of poor dietary choices) and have low effectiveness [29]. Our results suggest that educational strategies may not be as needed as direct strategies to modulate risk-taking, as obese individuals adequately appraise the consequences of choices but are still keen to take risks under certain circumstances. Findings should also be appraised in light of potential limitations. Firstly, measures of actual food intake behavior were not recorded, so the interpretation of a possible link between performance on the risky decision-making task and choosing highly palatable food can only be done so speculatively. In addition, although it was not an aim of the present study, we did not found differences between groups in SR. The inability to reproduce previous findings pointing to a curvilinear association between BMI and SR [17] could be due to a lack of statistical power in our sample to test self-report measures. Nonetheless, it should be stressed that the selection process of our sample was particularly rigorous when controlling for sociodemographic variables and excluding the possibility of comorbidities. This gives added value to the results obtained from our neuropsychological tasks; namely, obesity is uniquely associated with risky choices under conditions of probable gain and minimal loss, akin to real-life food choices.

## Supporting Information

S1 File Data file. (SAV)
